# Recent advances of silver nanoparticle-based polymer nanocomposites for biomedical applications

**DOI:** 10.1039/d4ra08220f

**Published:** 2025-03-19

**Authors:** Mohammad Harun-Ur-Rashid, Tahmina Foyez, Suresh Babu Naidu Krishna, Sudhakar Poda, Abu Bin Imran

**Affiliations:** a Department of Chemistry, International University of Business Agriculture and Technology (IUBAT) Sector 10, Uttara Model Town Dhaka 1230 Bangladesh mrashid@iubat.edu; b Department of Pharmacy, School of Life Sciences, United International University United City, Madani Ave Dhaka 1212 Bangladesh tahmina@pharmacy.uiu.ac.bd; c Institute for Water and Wastewater Technology, Durban University of Technology P. O. Box 1334 Durban 4000 South Africa sureshk@dut.ac.za; d Department of Biotechnology, Acharya Nagarjuna University Andhra Pradesh India sudhakarp@anu.ac.in; e Department of Chemistry, Bangladesh University of Engineering and Technology (BUET) Dhaka 1000 Bangladesh abimran@chem.buet.ac.bd

## Abstract

Silver nanoparticle–polymer nanocomposites (AgNP–PNCs) represent a transformative advancement in biomedical material science, integrating the potent antimicrobial properties of AgNPs with the structural versatility of polymer matrices. This synergy enables enhanced infection control, mechanical stability, and controlled drug delivery, making these nanocomposites highly suitable for applications such as wound healing, medical coatings, tissue engineering, and biosensors. Recent progress in synthesis and functionalization has led to greater control over particle morphology, dispersion, and stability, optimizing AgNP–PNCs for clinical and translational applications. However, challenges related to cytotoxicity, long-term stability, immune response, and scalability persist, necessitating systematic improvements in surface functionalization, hybridization strategies, and biocompatibility assessments. This review critically evaluates the latest advancements in AgNP–PNC development, focusing on their functionalization techniques, regulatory considerations, and emerging strategies to overcome biomedical challenges. Additionally, it discusses preclinical and translational aspects, including commercialization barriers and regulatory frameworks such as FDA and EMA guidelines, ensuring a comprehensive outlook on their clinical feasibility. By bridging the gap between innovation and practical application, this review investigates the transformative potential of AgNP–PNCs in advancing next-generation biomedical materials.

## Abstract 2

Silver nanoparticle (AgNP)-polymer nanocomposites offer enhanced antimicrobial properties for biomedical applications, including medical devices and implants. Stability and biocompatibility remain key challenges to apply AgNPs in biomedical fields.

## Graphical abstract

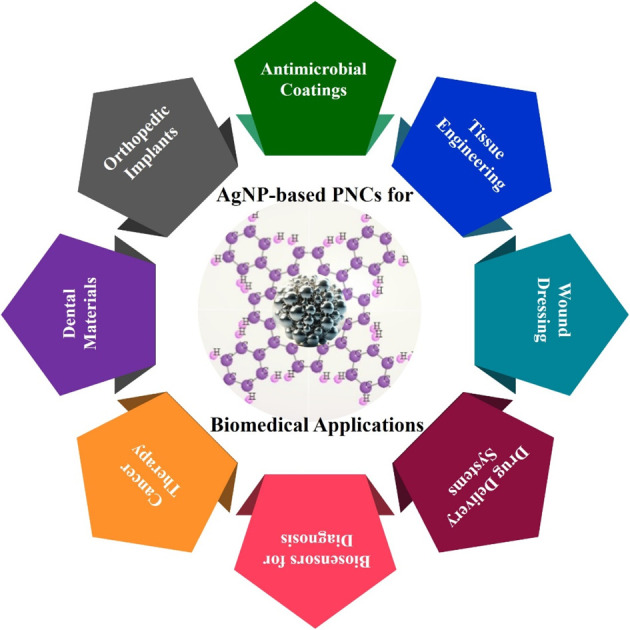

## 1. Introduction

In recent years, nanotechnology has revolutionized multiple scientific and clinical fields, enabling the development of advanced materials with unprecedented properties, which offer significant potential in addressing complex biomedical challenges.^1^ Among these materials, silver nanoparticles (AgNPs) have garnered considerable attention due to their powerful antimicrobial, optical, and electrical properties, making them particularly valuable in biomedical applications.^2,3^ However, stability, controlled release, and cytotoxicity challenges have limited their direct clinical use. To overcome these limitations, integrating AgNPs within polymer matrices to form AgNP–polymer nanocomposites (AgNP–PNCs) has emerged as a transformative strategy, combining the bioactive properties of silver with the versatility, stability, and biocompatibility of polymers.^4^ The AgNP–PNCs provide a controlled and sustained release of silver ions while mitigating the cytotoxic effects typically associated with free AgNPs.^5^ The polymer matrix is a stabilizing medium, reducing nanoparticle aggregation, improving biocompatibility, and allowing functional modifications to tailor the composite's mechanical, chemical, and biological properties for specific biomedical applications.^6^ AgNP–PNCs are, therefore, promising candidates for infection control, regenerative medicine, and targeted drug delivery, with applications from wound dressings and implantable medical devices to tissue scaffolds and biosensors.^7^ Despite these advantages, the widespread biomedical adoption of AgNP–PNCs remains hindered by several critical challenges. The biocompatibility and cytotoxicity of AgNPs remain a major concern, as their biological effects are strongly dependent on particle size, shape, surface chemistry, and silver ion release kinetics.^8,9^

Additionally, achieving long-term stability and uniform dispersion of AgNPs within polymer matrices is a key challenge, as agglomeration can significantly reduce their functionality. The scalability and reproducibility of AgNP–PNC synthesis at an industrial level also present significant obstacles, requiring the development of cost-effective, eco-friendly, and standardized fabrication techniques. The regulatory approval for AgNP-based biomedical materials remains complex, with concerns over their long-term toxicity, environmental impact, and potential bacterial resistance. This review provides an in-depth analysis of AgNP–PNCs, focusing on their synthesis techniques, functionalization strategies, biomedical applications, and future perspectives. We critically examine the latest advancements in AgNP–PNC design, explore strategies for enhancing their stability, biocompatibility, and antimicrobial efficiency, and discuss the regulatory landscape governing their biomedical use. Finally, we highlight the current limitations and emerging innovations that will shape the future of AgNP–PNCs in clinical and commercial applications.

## 2. Background and scope of the review

AgNPs have attracted substantial interest in biomedical research due to their potent antimicrobial, anti-inflammatory, and regenerative properties, making them highly suitable for various medical applications such as wound care, surgical coatings, and drug delivery.^10^ Integrating AgNPs into polymer matrices to form AgNP–PNCs enhances stability, controls silver ion release, and reduces cytotoxicity while maintaining strong antimicrobial efficacy.^11^ However, despite the growing number of studies on AgNPs and polymer nanocomposites, a comprehensive review focusing specifically on AgNP–PNCs for biomedical applications remains limited.

Existing literature has mainly focused on individual components-either AgNPs alone or polymer-based nanocomposites—but often lacks a systematic integration of advancements in AgNP–PNC synthesis, functionalization strategies, and application-specific modifications.^12^ While some studies have highlighted the use of AgNPs in commercial biomedical products, such as wound dressings, orthopedic implants, and biosensors, they often overlook critical challenges related to biocompatibility, long-term stability, cytotoxicity, and clinical translation.^13^ A key challenge in biomedical applications of AgNP–PNCs is biocompatibility and cytotoxicity, which depend on particle size, shape, concentration, and silver ion release kinetics, requiring optimized formulations for safe use. Stability and scalability also pose significant concerns, as nanoparticle agglomeration and degradation within polymer matrices can affect performance and safety. Fabrication techniques must be eco-friendly, cost-effective, and scalable to support large-scale production. Additionally, regulatory approval remains complex due to uncertainties regarding toxicity, environmental impact, and bacterial resistance, necessitating extensive preclinical and clinical studies.

This review aims to bridge existing gaps by providing a systematic and in-depth analysis of AgNP–PNCs, focusing on key aspects such as advancements in synthesis and functionalization, application-specific enhancements, and challenges in clinical translation. It critically evaluates various synthesis methods, including *in situ* polymerization, electrospinning, and solution blending, assessing their impact on biomedical performance. The review further explores the role of AgNP–PNCs in antimicrobial coatings, drug delivery, tissue engineering, and biosensing technologies, highlighting modifications that enhance their efficacy. Additionally, it discusses biocompatibility concerns, regulatory hurdles, and emerging innovations that could facilitate their safe and effective use in medical applications. A structured approach is adopted to systematically examine the fundamental properties of AgNPs, synthesis strategies, biomedical applications, and regulatory considerations. By integrating recent advancements with critical challenges, this review serves as a comprehensive resource for researchers, material scientists, and biomedical engineers, providing valuable insights into the development and clinical application of AgNP–PNCs.

## 3. Fundamentals of AgNPs

Silver has been valued for centuries due to its antimicrobial properties, conductivity, and chemical stability, making it an essential material in various applications, ranging from medicine and electronics to catalysis and water purification. The use of colloidal AgNPs in commercial applications dates back to 1897 with the introduction of Collargol, followed by stabilized forms such as Argyrol in the early 20th century. By 1954, colloidal silver was registered as a biocide under the U.S. Federal Insecticide, Fungicide, and Rodenticide Act (FIFRA), and it continues to be widely utilized in antimicrobial formulations.^14^ With the advent of nanotechnology, AgNPs have gained significant interest due to their unique size-dependent properties, enabling enhanced antimicrobial efficiency, tunable optical characteristics, and catalytic activity. Recent research has expanded AgNP applications beyond traditional uses to PNCs, leading to advanced biomedical solutions such as infection-resistant coatings, wound dressings, tissue scaffolds, biosensors, and targeted drug delivery systems.

### 3.1. Unique properties of AgNPs

AgNPs exhibit distinct physicochemical properties that differ from bulk silver due to their high surface area-to-volume ratio, surface plasmon resonance (SPR), and quantum confinement effects. These properties contribute to their enhanced electrical conductivity, catalytic efficiency, and biological interactions, making them suitable for diverse biomedical applications.^15^

#### 3.1.1. Optical and electrical properties

AgNPs demonstrate SPR, wherein conduction electrons resonate at specific wavelengths of incident light, leading to size- and shape-dependent optical characteristics. This phenomenon is widely exploited in biosensors, imaging techniques, and photothermal therapy. Due to their high electrical conductivity, AgNPs are also used in flexible electronics, conductive inks, and smart biomedical devices.^16^ AgNPs can be functionalized with superparamagnetic coatings, such as iron oxide (Fe_3_O_4_), to enhance their applications in targeted drug delivery and Magnetic Resonance Imaging (MRI), a non-invasive imaging technique widely used for high-resolution visualization of soft tissues and anatomical structures. This magnetic functionalization allows AgNPs to be manipulated using external magnetic fields, improving precision and controlled localization in biomedical applications. However, adding magnetic coatings can modify the optical, electrical, and catalytic properties of AgNPs, which may impact their performance in sensing, diagnostics, or antimicrobial applications. To mitigate these challenges, optimized coating thickness and material composition must be carefully designed to preserve the multifunctionality of AgNP-based nanocomposites.

#### 3.1.2. Antimicrobial and biocidal mechanisms

AgNPs are among the most effective antimicrobial agents, exhibiting broad-spectrum activity against bacteria, fungi, and viruses. Their antimicrobial mechanisms involve:

• Cell membrane disruption – AgNPs bind to bacterial membranes, causing structural damage and increased permeability.

• Reactive Oxygen Species (ROS) generation – AgNPs induce ROS production, leading to oxidative stress, DNA fragmentation, and protein dysfunction in microbial cells.

• Protein and enzyme inhibition – silver ions interact with thiol (–SH) groups in bacterial proteins, disrupting essential enzymatic functions and preventing microbial growth.

These properties make AgNPs highly effective in antibacterial wound dressings, orthopedic implants, and medical coatings, providing long-term infection control without antibiotic resistance concerns.

#### 3.1.3. Biomedical functionalization: magnetic and hybrid coatings

Recent advancements in magnetic-functionalized AgNPs have expanded their biomedical utility, particularly in MRI contrast enhancement and targeted drug delivery. Incorporating superparamagnetic coatings, such as Fe_3_O_4_, allows AgNPs to be manipulated by external magnetic fields, improving precision in diagnostics and therapeutics. However, such modifications can alter AgNPs' optical and electrical properties, necessitating optimized coating thickness and material composition to maintain their multifunctionality. Hybrid AgNP–polymer systems integrating biodegradable matrices further enhance their biocompatibility and controlled release capabilities, making them ideal for tissue engineering, bioimaging, and cancer therapy.

### 3.2. Synthesis strategies for AgNPs

AgNP synthesis methods are broadly classified into top-down and bottom-up approaches, each offering distinct advantages for controlling particle size, shape, and stability.

#### 3.2.1. Top-down synthesis: physical disintegration of bulk silver

Top-down approaches involve breaking down bulk silver into nanoparticles through mechanical, thermal, or laser-based methods. Common techniques include:

• Laser ablation – a high-energy laser pulse vaporizes bulk silver into nanosized particles, ensuring precise control over size and purity.^17^

• Ball milling – mechanical force reduces silver into nanoscale particles, often combined with surfactants to prevent agglomeration.

• Sputtering and lithography – used in microfabrication for thin-film deposition, creating patterned AgNP layers for biosensors and electronic devices.^18^

While top-down methods provide high purity nanoparticles, they require significant energy input and lack precise control over size uniformity.

#### 3.2.2. Bottom-up synthesis: chemical and biological routes

Bottom-up approaches rely on chemical reduction or biological synthesis to assemble AgNPs from precursor molecules.^19^

• Chemical reduction – the most common method, utilizing reducing agents (*e.g.*, sodium borohydride, citrate) to convert AgNO_3_ into AgNPs.

• Green synthesis – an eco-friendly approach using plant extracts, bacteria, or fungi as bioreducing agents, producing AgNPs with enhanced biocompatibility.

• Microbial and enzymatic synthesis – exploiting biological pathways in bacteria or fungi to create AgNPs with natural capping agents for stability.

Biogenic AgNP synthesis is gaining interest due to its low toxicity, sustainability, and tunable surface functionalization, making it highly suitable for biomedical and environmental applications.

## 4. Optimization of AgNPs for the fabrication of AgNP-based PNC

The AgNP-based PNC enhances their mechanical, electrical, and biological attributes, making them ideal for biomedical use. However, optimizing AgNPs for safe and effective application in PNCs requires precise control over nanoparticle size, shape, dispersion, stability, and biocompatibility. Additionally, ensuring scalability for industrial production is essential for their widespread clinical use.

### 4.1. Optimization of synthetic methods

The synthesis method significantly influences the properties of AgNPs, impacting their integration into PNCs. Common techniques include chemical, electrochemical, photochemical, and green synthesis methods.^20^ Chemical reduction is widely preferred due to its cost-effectiveness, scalability, and ability to control particle size and shape by adjusting reducing agents, stabilizers, temperature, and reaction time. Electrochemical and photochemical reductions, although offering better precision, are less commonly employed due to their complexity and high costs. Recent advancements in green synthesis, using plant extracts, bacteria, or fungi, have emerged as eco-friendly alternatives, improving biocompatibility for medical applications. Table 1 provides a comparative overview of various synthesis methods for AgNPs and their integration into PNCs.

**Table 1:** Summary of AgNP synthesis methods, optimization parameters, polymer types, applications, and pros and cons

Synthesis method	Optimization parameters	Polymer type	Applications	Pros	Cons
Chemical reduction	Reducing agent type, stabilizer, temperature, reaction time, pH	Polyvinyl alcohol (PVA), poly(ethylene oxide) (PEO), polyethylene glycol (PEG), chitosan	Antimicrobial coatings, wound dressings, drug delivery	Cost-effective, scalable, easy size control	Requires toxic chemicals, stability issues
Electrochemical reduction	Voltage, electrolyte type, current density, reaction time	Polymethyl methacrylate (PMMA), poly(caprolactone) (PCL), PEG	Biosensors, orthopedic implants, drug carriers	High purity, precise control of shape/size	Expensive equipment, requires conductive polymer
Photochemical reduction	Light intensity, wavelength, irradiation time, solvent medium	PVP, polylactic acid (PLA), chitosan	Antimicrobial coatings, photodynamic therapy, wound dressings	Eco-friendly, avoids harsh chemicals	Requires precise light source, limited scalability
Laser ablation	Laser power, pulse duration, wavelength, target material	PLA, poly(dimethylsiloxane) PDMS, PU	Biosensors, dental materials, tissue engineering	No chemical reagents required, high purity	High cost, requires specialized equipment
Green synthesis (biogenic/plant-based)	Plant extract concentration, pH, temperature, reaction time	Alginate, chitosan, cellulose	Wound healing, tissue engineering, drug delivery	Environmentally friendly, non-toxic, biocompatible	Lack of reproducibility, longer reaction times
Microwave-assisted synthesis	Microwave power, reaction time, precursor concentration	PLA, PEG, PVA	Biosensors, antimicrobial coatings, drug delivery	Rapid synthesis, energy efficient, uniform particle distribution	Potential for overheating, polymer degradation
Sol–gel method	Solvent type, precursor concentration, hydrolysis time, drying temperature	PMMA, PEG, PEO	Bone tissue engineering, dental applications, implant coatings	High control over particle size, improved stability	Complex processing, long reaction time
Spray pyrolysis	Spray rate, solvent evaporation temperature, precursor concentration	PDMS, polyurethane (PU)	Antimicrobial coatings, biomedical sensors	Produces uniform nanoparticles, scalable	High-temperature process, risk of polymer degradation
Hydrothermal synthesis	Temperature, pressure, reaction time, precursor concentration	Chitosan, PVA, PCL	Tissue scaffolds, orthopedic implants, biosensors	High crystallinity, better biocompatibility	Requires high pressure, slow reaction time
*In situ* polymerization	Monomer ratio, reaction temperature, polymerization time	PEG, chitosan, PMMA	Drug delivery, tissue engineering, wound dressings	Direct nanoparticle–polymer integration, strong polymer–nanoparticle interactions	Requires precise reaction conditions, potential polymer degradation

### 4.2. Optimization of particle size and shape

The size and shape of AgNPs significantly influence their antimicrobial activity, toxicity, and biocompatibility within PNCs. Factors such as reducing agent concentration, stabilizer type, reaction temperature, and synthesis duration determine the final nanoparticle morphology.^21^ The smaller AgNPs exhibit enhanced antimicrobial properties but may pose higher cytotoxicity risks. Additionally, anisotropic shapes (*e.g.*, nanoplates, nanorods) offer unique plasmonic properties beneficial for biosensors and imaging applications. Precise control over these parameters ensures optimized AgNP performance in biomedical applications such as targeted drug delivery and wound healing.

### 4.3. Optimization of particle dispersity in polymer matrix

Uniform dispersion of AgNPs within the polymer matrix ensures consistent properties and avoids aggregation, which can compromise the material's mechanical and biological performance. Techniques such as ultrasonication, high-shear mixing, and melt compounding are commonly employed to achieve homogeneity. Aguiar *et al.*^22^ successfully demonstrated uniform silver dispersion in a PVA matrix using mechanical alloying, producing nanocomposites with antibacterial and hydrophilic properties. Optimizing dispersion techniques is particularly important for ensuring the longevity and efficacy of AgNP–PNCs in medical devices and coatings.

### 4.4. Optimization of particles' nanotoxicity and biocompatibility

Nanotoxicity remains a primary challenge in biomedical applications of AgNP–PNCs.^23^ The cytotoxicity of AgNPs varies depending on concentration, size, and surface chemistry. Surface functionalization strategies such as coating AgNPs with biocompatible polymers (*e.g.*, PEG, chitosan) are employed to minimize adverse effects. Additionally, comprehensive *in vitro* and *in vivo* toxicity assessments help establish safe dosage levels for clinical applications. Recent studies have shown that AgNP concentrations below 10 ppm exhibit minimal cytotoxicity while retaining antimicrobial efficacy. Enhancing AgNP stability and optimizing release kinetics further ensure biocompatibility in biomedical applications.

### 4.5. Optimization of particles' stability

AgNP stability is crucial, as aggregation and oxidation can degrade their antimicrobial and conductive properties over time. Surface functionalization and stabilizers such as surfactants or polymers can effectively prevent aggregation. Retout *et al.* developed a method using calix[4]arenetetradiazonium salt to enhance nanoparticle stability by preventing surface polymerization.^24^ Ensuring stable dispersion within the polymer matrix is vital for maintaining the long-term efficacy of AgNP–PNCs in medical applications.

### 4.6. Optimization of mechanical characteristics

The mechanical properties of AgNP–PNCs must be optimized to enhance durability and flexibility for biomedical applications. Excessive AgNP loading can lead to particle aggregation, reducing mechanical strength. Deka *et al.* developed a stable PVA–guar gum hydrogel with AgNPs (10–20 nm), achieving a 74% increase in tensile strength and improved swelling capacity.^25^ The optimization of polymer compatibility and nanoparticle dispersion give superior mechanical performance, making these materials suitable for applications in tissue scaffolds and wound dressings.

### 4.7. Optimization of production scalability

To ensure large-scale commercial viability, AgNP–PNC synthesis must be scalable, cost-effective, and eco-friendly. Various processing techniques such as melt blending, solution blending, and *in situ* polymerization allow efficient integration of AgNPs into polymer matrices while preserving nanoparticle properties. Nogueira *et al.* demonstrated a scalable AgNP production approach using a semi-batch reactor, ensuring consistent quality across reactor sizes and confirming antibacterial properties through UV-vis, TEM, and XRD analyses.^26^ These scalable methods are essential for transitioning AgNP–PNCs from laboratory research to industrial production for medical applications. By optimizing synthesis parameters, nanoparticle properties, dispersion techniques, biocompatibility, stability, mechanical characteristics, and scalability, AgNP–PNCs can be effectively tailored for biomedical applications. Future research should further refine these optimization strategies to improve safety, efficacy, and commercial feasibility in clinical settings.

## 5. Prospective materials for fabricating AgNPs–PNCs

The selection of materials for AgNP–PNCs plays a crucial role in determining their biocompatibility, mechanical properties, antimicrobial efficacy, and functional stability for biomedical applications. Both natural and synthetic polymers, as well as inorganic and carbon-based materials, serve as effective matrices for incorporating AgNPs, enabling the development of advanced nanocomposites with tailored properties for applications such as wound dressings, implant coatings, biosensors, and drug delivery systems.

### 5.1. Natural polymers

Natural polymers have been widely used in AgNP–PNC fabrication due to their biodegradability, biocompatibility, and ability to interact with biological systems. Chitosan, a polysaccharide derived from chitin, exhibits antimicrobial and anti-inflammatory properties, making it ideal for wound healing and drug delivery applications.^27^ Gelatin, a collagen-derived biopolymer, enhances the mechanical stability of nanocomposites and is commonly used in pharmaceuticals and tissue engineering.^28^ Alginate, a naturally occurring anionic polymer, is extensively utilized for wound dressings and tissue scaffolds due to its ability to form hydrogels in the presence of calcium ions.^29^ Cellulose-based materials, known for their strength and biodegradability, are stabilizing matrices in AgNP–PNCs, making them useful for coatings, films, and biomedical applications.^30^ Starch-based AgNP–PNCs have demonstrated promising results as biodegradable carriers for drug delivery, providing enhanced stability and controlled release properties.^31^ Silk fibroin, derived from silkworms, is increasingly being explored in regenerative medicine and tissue engineering, owing to its unique self-assembling nature and strong biocompatibility.^32^ Hyaluronic acid is essential in moisture retention and wound healing, making it highly effective for hydrogels and injectable drug carriers.^33^ Collagen, an essential structural protein, is widely employed in tissue scaffolding and regenerative medicine.^34^ Carrageenan, a sulfated polysaccharide obtained from red seaweed, has applications in biomedical coatings, antiviral formulations, and anti-inflammatory therapies.^35^

### 5.2. Synthetic polymers

Synthetic polymers such as PDMS, PEG, PVA, poly(lactic-*co*-glycolic acid) (PLGA), PEO, PLA, PU, PMMA, and PCL are widely used in AgNP–PNCs for biomedical applications due to their versatility, stability, and compatibility with medical uses. PDMS is highly flexible, biocompatible, and used in microfluidics, tissue engineering, and implantable devices.^36^ PEG is extensively applied in hydrogels and targeted imaging, enhancing drug solubility and controlled release properties^37^ provides excellent biocompatibility and mechanical properties, making it suitable for wound dressings and medical coatings.^38^ PLGA, also biodegradable, is commonly used in drug delivery and tissue engineering, while PEO's water solubility aids in similar applications.^39^ PU is frequently used in implantable medical devices and prosthetics due to its high elasticity and resistance to mechanical stress.^40^ PMMA, a well-established material in ophthalmology and dentistry, offers superior tissue compatibility and structural integrity, making it a preferred choice for intraocular lenses, bone cement, and dental implants.^41^

### 5.3. Inorganic materials

Inorganic materials serve as structural reinforcements in AgNP–PNCs, enhancing mechanical properties, stability, and bioactivity. Silica nanoparticles provide high surface area and excellent stability, making them suitable for biosensors, targeted drug delivery, and imaging applications.^42^ Titanium dioxide (TiO_2_), widely recognized for its biocompatibility and photocatalytic properties, is incorporated into AgNP–PNCs for antimicrobial coatings, dental materials, and orthopedic implants.^43^ Hydroxyapatite (HA), structurally similar to natural bone, is a key component in bone grafts, implants, and tissue engineering due to its ability to promote osseointegration and bioactivity.^44^ Zinc oxide (ZnO) exhibits antimicrobial, UV-blocking, and piezoelectric properties, making it useful for wound dressings, biosensors, and antimicrobial coatings.^45^ Gold nanoparticles (AuNPs) have been integrated into AgNP–PNCs to enhance plasmonic and photothermal properties, particularly for cancer therapy, imaging, and antimicrobial applications.^46^

### 5.4. Carbon-based materials

Carbon-based materials such as graphene, carbon nanotubes (CNTs), and carbon dots have gained significant interest in AgNP–PNC fabrication due to their high mechanical strength, electrical conductivity, and antimicrobial capabilities. Graphene provides a high surface area for AgNP immobilization, enhancing conductivity and stability in biosensors and drug delivery systems.^47^ CNTs, with their high aspect ratio and unique electronic properties, are being explored for targeted drug delivery and antimicrobial coatings. Carbon dots, due to their fluorescence properties and low cytotoxicity, have shown promise in bioimaging and cancer diagnostics.

## 6. Methods for fabricating AgNP–PNCs

Fabricating AgNP–PNCs involves various techniques influencing particle distribution, stability, and overall composite performance. The choice of fabrication method determines nanocomposite morphology, mechanical strength, and suitability for biomedical applications.

### 6.1. *In situ* polymerization

*In situ* polymerization allows for the direct synthesis of AgNPs within a polymer matrix, ensuring homogeneous nanoparticle distribution. This method provides enhanced mechanical properties and stability, making it suitable for implant coatings and drug carriers.^48^

### 6.2. Solution blending

Solution blending involves the physical mixing of AgNPs and polymers in a liquid solvent, ensuring uniform nanoparticle dispersion. This technique is commonly used for hydrogels, antimicrobial films, and biomedical coatings.^49^

### 6.3. Melt mixing

Melt mixing employs thermal and mechanical forces to disperse AgNPs into polymer matrices, resulting in strong interfacial interactions and improved mechanical strength. This approach is highly scalable, making it suitable for medical implants and prosthetic devices.^50^

### 6.4. Electrospinning

The electrospinning technique for creating AgNP–PNCs involves emitting a continuous jet of a charged polymer solution that is electrostatically drawn onto a collector.^51^ This method produces an ultrafine fibrous structure that embeds AgNPs within the polymer matrix. Electrospinning offers precise control over the dimensions and morphology of the fibers, which can be customized for specific biomedical applications.

### 6.5. Layer-by-layer assembly

The creation of AgNP–PNCs *via* the layer-by-layer assembly technique involves the sequential deposition of alternating layers of a polymer and AgNPs onto a substrate.^52^ This method produces a multi-layered thin film nanocomposite characterized by precisely controlled thickness and uniform distribution of AgNPs within the polymer matrix. The layer-by-layer assembly provides a sophisticated means to fabricate nanocomposites with considerable structural and functional complexity, which can be specifically tailored for distinct biomedical applications.

### 6.6. *In situ* reduction

The synthesis of AgNP–PNCs *via in situ* reduction involves the chemical reduction of a silver precursor in the presence of a polymer matrix to form AgNPs directly within the matrix.^53^ This method leverages chemical reduction to achieve the nanocomposite material. The *in situ* reduction process requires meticulous control over various parameters, including the concentration of the silver precursor and the reaction time, to ensure a well-dispersed and stable nanocomposite.

### 6.7. Ultrasonication

Ultrasonication employs high-frequency sound waves to enhance AgNP dispersion within polymers, improving mechanical properties and antimicrobial performance in medical coatings and wound dressings.^54^

### 6.8. Spray pyrolysis

Spray pyrolysis produces AgNP–PNCs by depositing a mist of precursors onto a heated substrate, yielding uniform coatings with high thermal and chemical stability.^55^

### 6.9. Sol–gel method

The sol–gel method for fabricating AgNP–PNCs in biomedical applications involves preparing a colloidal AgNP solution and mixing it with a polymer precursor to form a gel. This gel is dried and heated to induce polymerization, creating a stable nanocomposite. Effective production requires precise control of precursor concentration and heating rate to ensure uniformity and stability, enhancing the composite's thermal and mechanical properties. Bagheri *et al.* demonstrated this method by synthesizing a silica–polydiphenylamine–AgNP nanocomposite (Ag–SiO_2_–PDPA) using butanethiol-capped AgNPs, where SiO_2_ spheres stabilized the AgNPs through surface adsorption.^56^

### 6.10. Microwave-assisted synthesis

**Fig. 1:**
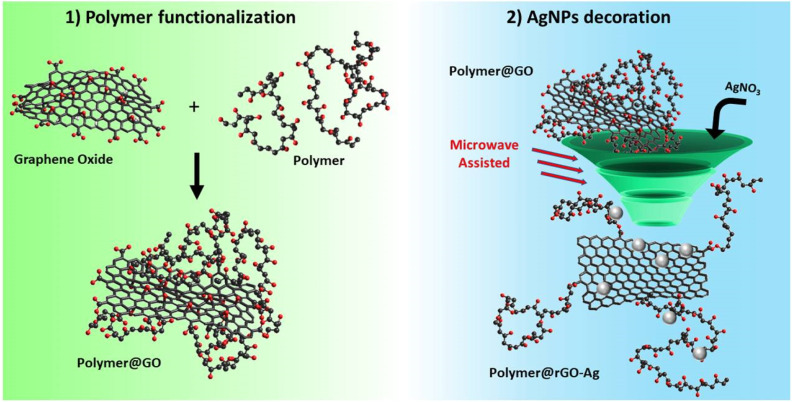
A diagram illustrating the process of polymer functionalization and AgNPs decoration. Reproduced with permission.^57^ Copyright 2020 by the authors. Licensee MDPI, Basel, Switzerland.

**Fig. 2:**
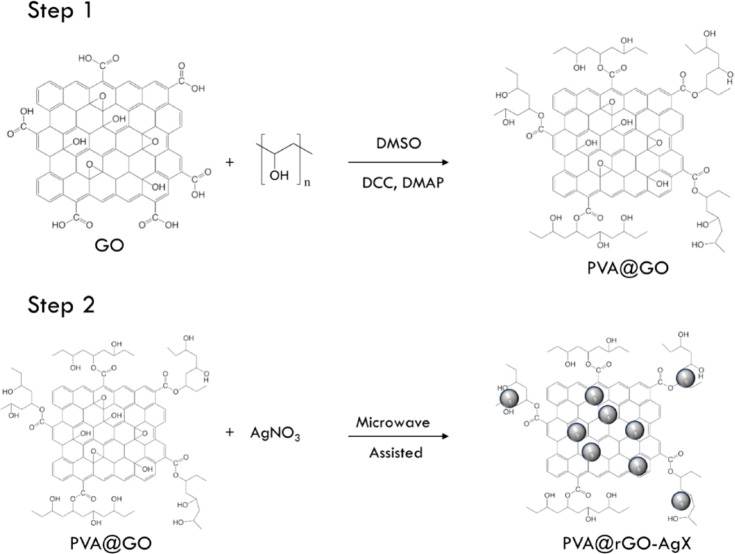
A diagram illustrating the process of creating hybrid systems made of PVA GO and silver denoted as PVA@rGO–AgX. Reproduced with permission.^57^ Copyright 2020 by the authors. Licensee MDPI, Basel, Switzerland.

Microwave-assisted synthesis of AgNP–PNCs for biomedical applications involves subjecting a polymer and AgNP precursor mix to microwave irradiation, rapidly reducing the precursor and embedding AgNPs within the polymer matrix. This efficient method yields high homogeneity and reproducibility, with enhanced thermal and electrical conductivity, making it ideal for biomedical use. Nicosia *et al.* developed antimicrobial AgNP–graphene nanocomposites (NanoHy-GPS) using microwave irradiation without reductants or surfactants, achieving controlled AgNP loading (5–87%) and uniform particle distribution (5–12 nm)^57^ (Fig. 1). To esterify graphene oxide (GO), PVA hydroxyl groups were used (Fig. 2, step 1), followed by a direct microwave-assisted silver ion reduction (Fig. 2, step 2).

### 6.11. Hydrothermal synthesis

Hydrothermal synthesis generates well-dispersed AgNPs within polymer matrices under high-pressure conditions, ensuring enhanced biocompatibility and long-term stability.^58^Table 2 provides a comparative overview of AgNP–PNC synthesis techniques, including their reaction conditions, polymer compatibility, advantages, disadvantages, and biomedical applications. It facilitates an informed selection of synthesis approaches based on the desired nanocomposite properties and specific medical applications.

**Table 2:** Comparative summary of AgNP–PNC synthesis procedures, reaction conditions, polymer types, advantages, disadvantages, and applications

Synthesis method	Reaction conditions	Polymer type	Advantages	Disadvantages	Applications
*In situ* polymerization	Controlled temperature, monomer–polymer interaction, catalyst-assisted reaction	PEG, PMMA, chitosan	Strong nanoparticle–polymer interaction, good dispersion, stable composite	Requires precise reaction conditions, potential polymer degradation	Drug delivery, tissue engineering, wound dressings
Solution blending	Polymer and AgNPs dissolved in solvent, mixed under controlled conditions	PVA, PEG, PU	Simple, cost-effective, scalable	Risk of AgNP aggregation, solvent removal required	Antimicrobial coatings, medical textiles, drug carriers
Melt mixing	High-temperature polymer melting, mechanical shear force for AgNP dispersion	PLA, PMMA, PDMS	No need for solvents, good dispersion, high mechanical properties	High temperature can degrade certain polymers and AgNPs	Orthopedic implants, antimicrobial coatings
Electrospinning	Polymer and AgNPs dissolved in a solution, electrostatic force used for nanofiber formation	PLA, PVA, chitosan	Produces ultrafine nanofibers, tunable porosity, high surface area	Requires optimized conditions, limited scalability	Wound dressings, scaffolds, filtration membranes
Layer-by-layer assembly	Sequential deposition of AgNPs and polymer layers on a substrate	PEO, PVP, chitosan	Precise thickness control, homogeneous nanoparticle distribution	Time-consuming, requires specialized equipment	Biosensors, antimicrobial coatings, tissue engineering
*In situ* reduction	Chemical reduction of silver precursor within polymer matrix	PVP, PEG, PMMA	Homogeneous nanoparticle distribution, good stability	Requires careful control of reaction parameters	Drug delivery, wound dressings, antibacterial coatings
Ultrasonication	High-frequency sound waves applied to AgNP–polymer mixture	PVA, chitosan, PEO	Good dispersion, prevents aggregation	Requires optimization to avoid polymer degradation	Biomedical coatings, antimicrobial films
Spray pyrolysis	Mist of AgNP–polymer precursor solution deposited on a heated substrate	PDMS, PU	Produces uniform AgNP–polymer coatings	High-temperature process, risk of polymer degradation	Antimicrobial medical devices, bioactive coatings
Sol–gel method	Hydrolysis and polymerization of precursors in solution	PMMA, PEG, PVA	High stability, precise control of AgNP size	Long processing time, requires careful pH and temperature control	Dental materials, tissue scaffolds, orthopedic applications
Microwave-assisted synthesis	Microwave irradiation applied to AgNP–polymer mixture	PLA, PEG, PVA	Rapid synthesis, energy efficient, uniform AgNP dispersion	Potential for overheating, polymer degradation	Drug delivery, antimicrobial textiles
Hydrothermal synthesis	High-pressure, high-temperature reaction in sealed vessel	Chitosan, PVA, PCL	Uniform AgNP distribution, biocompatible, good crystallinity	Requires specialized equipment, slow reaction time	Bone tissue engineering, biosensors, wound dressings

## 7. AgNP-based PNC

A composite material combines substances with distinct physical and chemical properties at a macroscopic level, yielding unique properties unattainable by individual components. Composites consist of a matrix, typically the continuous phase, and reinforcements like particles, fibers, or layers. Recent advancements in nanocomposites, with features such as toughness nearly 1000 times greater than bulk counterparts, leverage nanoscale dimensions for high surface-to-volume ratios, enhancing interactions within the matrix and yielding multifunctional materials.^59^ PNCs containing 1–50 nm nanoparticles capitalize on the nanoscale's distinct physical and chemical properties. The polymer matrix in PNCs aids in binding, chemical protection, and orientation of nanofillers. AgNPs, known for conductivity, stability, catalytic activity, and optical properties, are commonly included in PNCs, enhancing performance and stability by acting as ion-capping agents.^60^

The polymer matrix is an effective host for incorporating nanoparticles and controls nucleation to prevent excessive particle growth. Incorporating Ag nanoparticles significantly enhances the performance of the composite.^61^ AgNPs, which can be applied as coatings or additives, are used on various types of catheters, including PU ventricular catheters, commercially available under the brand names Silverline® and ON-Q SilverSoaker™.^62^ AgNPs are also present in hand gels^63^ and paints,^64^ providing durable antibacterial, antiviral, and antifungal properties. However, AgNPs alone face limitations due to high surface reactivity, which causes aggregation and reduces antimicrobial efficacy. To overcome this, researchers have focused on immobilizing AgNPs onto solid supports, with polymers as favored templates for easy incorporation, dispersion, and controlled synthesis. Silver–PNCs offer tailored antimicrobial coatings, sheets, or films that meet specific application needs, with natural polymers often preferred for their abundance, biocompatibility, and biodegradability.

### 7.1. AgNP-based natural PNC

AgNP-based biocidal coatings are crafted with biocompatible, non-toxic natural polymers like chitosan, alginate, and hyaluronic acid to tackle implant rejection, inflammation, and toxicity. AgNPs enhance chitosan's durability and antimicrobial properties. Nanotechnology increasingly favors eco-friendly natural polymers and nanoscale fillers to create bionanocomposites, especially AgNP composites. These natural polymer–AgNP composites improve mechanical, optical, and electrical properties, with polymers stabilizing AgNPs to prevent precipitation and agglomeration. AgNP-based natural PNCs can be synthesized using various methods, including mechanical mixing, *in situ* reduction of silver salts, and *in situ* polymerization.^65^ Alternative reduction methods, such as microwave and radiochemical techniques, are also employed.^66^ There have been numerous studies published on PNCs fabricated with a combination of AgNPs and natural polymers such as chitosan,^67^ gelatin ^68^, cellulose,^69^ collagen,^70^ alginate,^71^ starch,^72^ hyaluronic acid,^73^ silk fibroin,^74^ and carrageenans.^75^

### 7.2. AgNP-based synthetic PNC

Artificial synthetic polymers are nearly as diverse as natural ones and have rapidly gained use in medical applications, such as synthetic sutures made from polyesters and polyamides.^76^ These polymers are preferred for their customizable physical and chemical properties, often offering superior structural and mechanical strength compared to natural polymers. Their carbon-based composition allows them to closely mimic biological tissues, enhancing targeted interactions within the body. Reactive groups enable biofunctionalization, improving biocompatibility. Both biodegradable and non-biodegradable types are used, with biodegradable variants designed to degrade naturally after fulfilling their purpose, avoiding the need for surgical removal.^77^ Ideally, biodegradable synthetic polymers are designed to remain in the body only for their functional necessity and then degrade without further surgical intervention.^78^

## 8. Biomedical applications of AgNP–PNCs

**Fig. 3:**
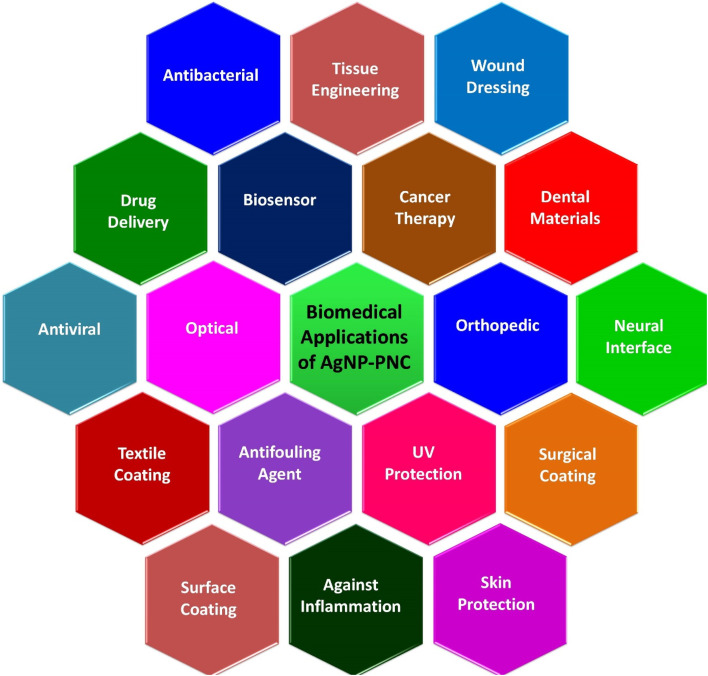
Prospective and multipurpose biomedical applications of AgNP–PNCs.

AgNP–PNCs are promising materials in biomedical research due to their unique properties. They possess strong antimicrobial effects against bacteria, viruses, and fungi, making them ideal for wound dressings and infection control in medical devices. Additionally, their anti-inflammatory properties support potential use in treating inflammatory conditions. With biocompatibility and easy functionalization, AgNP–PNCs are also attractive for targeted drug delivery and imaging applications, offering significant potential for innovative biomedical technologies, as summarized in Fig. 3.

### 8.1. Antimicrobial coatings for medical devices

**Fig. 4:**
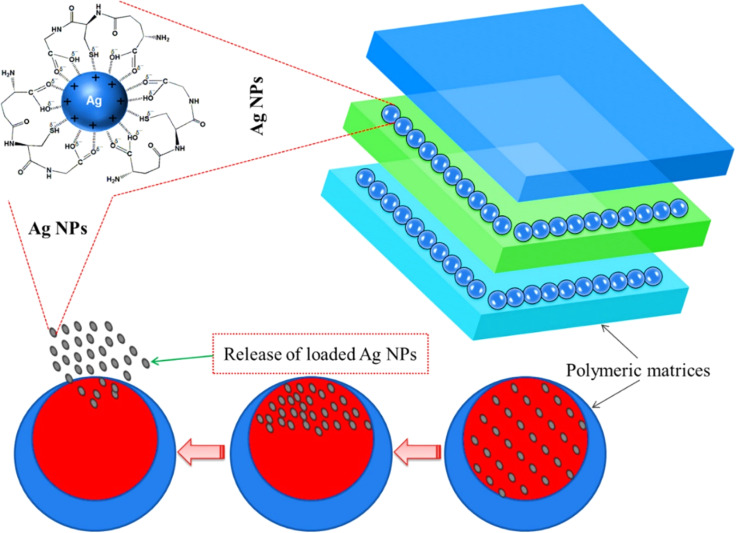
The schematic representation of the proposed release mechanism of AgNPs through polymeric matrices of AgNP–PNCs. Reproduced with permission.^79^ Copyright 2021 by Elsevier.

**Fig. 5:**
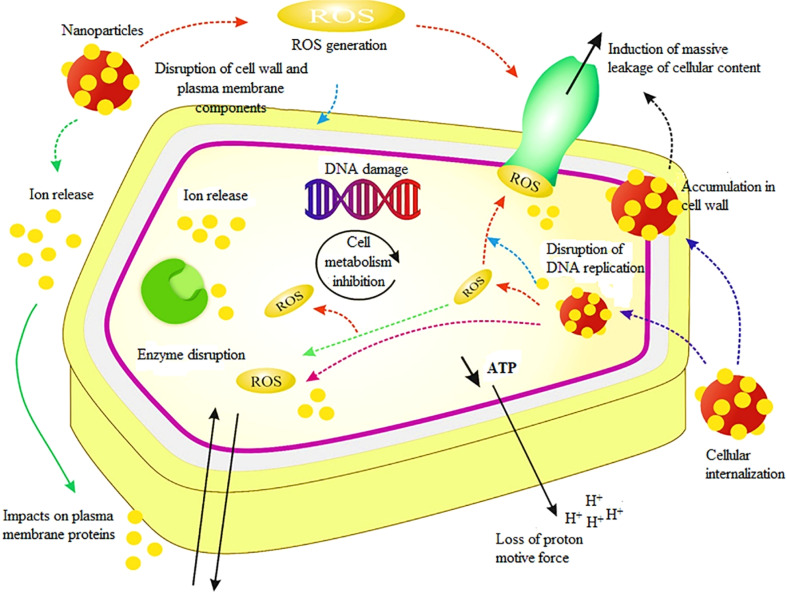
The schematic of antimicrobial actions of AgNPs–zeolite nanocomposite. Reproduced with permission.^79^ Copyright 2021 by Elsevier.

Antimicrobial refers to the capability to inhibit or eliminate microorganisms, including bacteria, viruses, fungi, and protozoans. Antimicrobial substances or agents are utilized to prevent or treat infections caused by these microorganisms. They are available in various forms, such as medications (including antibiotics, antivirals, and antifungals), disinfectants, sanitizers, and other products designed to control or eradicate harmful microorganisms from surfaces, objects, or living organisms. The antimicrobial properties of AgNP–PNCs play a crucial role in reducing the spread of infections and enhancing overall health and well-being. The process by which AgNPs are released from the polymer matrices and their antimicrobial action are depicted in Fig. 4 and 5, respectively.

Table 3 provides an overview of market-available AgNP–polymer nanocomposites used in wound care, surgical devices, orthopedic implants, dental hygiene, and infection control. It highlights the polymer type, manufacturer, application, and key functional features, offering insights into real-world implementations of AgNP–PNCs in biomedical fields.

**Table 3:** Commercially available AgNP–PNCs for biomedical applications

Product name	Manufacturer	Polymer type	Application	Key features
Acticoat™	Smith & Nephew [https://www.smith-nephew.com/en/health-care-professionals/products/advanced-wound-management/acticoat-global]	Polyethylene (PE)	Antimicrobial wound dressing	Broad-spectrum antibacterial action, sustained silver release
SilvaSorb®	Medline [https://www.medline.com/product/SilvaSorb-Silver-Antimicrobial-Wound-Gel/Z05-PF00181]	PVA hydrogel	Wound healing, burn care	Moist wound healing environment, controlled silver release
Aquacel® Ag^+^ Extra	Convatec [https://www.convatec.com/advanced-wound-care/aquacel-family-of-dressings/]	Sodium carboxymethylcellulose	Chronic wounds, pressure ulcers	Enhanced absorption, silver-based antimicrobial action
Silverlon®	Argentum Medical [https://www.silverlon.com/products/catheter-dressings]	Nylon-based	Surgical wound dressing, burn care	Silver-coated antimicrobial fabric, promotes healing
SilvrSTAT®	AsepticMD [https://silvrstat.com/wp-content/uploads/2022/07/Product_Insert.pdf]	Chitosan-based hydrogel	Wound care, surgical incisions	Fast-acting antimicrobial gel, biocompatible
SilverShield®	Argentum Medical [https://www.microban.com/antimicrobial-solutions/technologies/microban-silver-technology]	Silicone-based	Catheters, medical device coatings	Prevents biofilm formation, extended silver ion release
SilverCare® Toothbrush	SilverCare [https://www.norwex.com/p/adult-silver-care-toothbrush-with-refill-light-blue?srsltid=AfmBOoot4qTh-IDjso-ehzXzbbX4zfpGATAheRrTwuQLqb1bEWnyCSc2]	Polypropylene (PP)	Dental hygiene	Antibacterial protection, silver-infused bristles
Silverlon® Catheter Wraps	Argentum Medical [https://www.silverlon.com/products/catheter-dressings]	Polyester	Central venous catheter protection	Reduces catheter-associated infections

#### 8.1.1. Antibacterial coatings for medical devices

Hospital-acquired infections (HAIs) occur during treatment in healthcare settings like hospitals and clinics, caused by microorganisms such as bacteria, viruses, and fungi. Common HAIs include bloodstream infections, surgical site infections, urinary tract infections, and ventilator-associated pneumonia, leading to extended hospital stays, increased costs, and sometimes fatal outcomes. Prevention involves strict infection control, including hand hygiene, proper PPE use, and thorough cleaning of equipment and surfaces. Recent advancements focus on cost-effective technologies to prevent infections from long-term implants, emphasizing biomaterial surfaces that resist biofilm formation without harming mammalian cells or causing resistance. Biocompatible polymers with natural or modified antimicrobial properties—such as AgNPs—offer effective nosocomial infection prevention due to AgNPs' oligodynamic action and low human toxicity.^80^ Martorana *et al.* developed HA-DETA capped AgNPs using a green synthesis method, showing potential for controlling infections associated with medical devices, verified through various analytical techniques.^73^ Similarly, Bakhsheshi-Rad *et al.* improved Mg alloys for orthopedic use by electrospinning PLLA/GO–AgNP coatings, which enhanced corrosion resistance, strength, antibacterial efficacy, and cell growth. Although high levels of GO–AgNP co-incorporation slightly inhibited cell proliferation, the PLLA/GO–AgNP coatings present a promising solution for orthopedic applications.^81^

#### 8.1.2. Antiviral agents

AgNP–PNCs are promising antiviral materials, effective against viruses such as HIV, herpes simplex, and influenza. These composites could enhance personal protective equipment (PPE) like masks and gloves and medical devices like catheters by preventing viral infections. Additionally, they offer potential for air filtration systems to remove airborne viruses and are explored as topical or inhalation treatments for viral infections.^82^ Hasanin *et al.* developed a sustainable AgNP–PNC made of starch, oxidized cellulose, and ethyl cellulose, showing antibacterial, antifungal, and antiviral properties.^83^ This composite inhibited bacterial strains like *E. coli* and *S. aureus* and demonstrated antifungal and dose-dependent antiviral effects against herpes simplex, adenovirus, and Coxsackie B virus. Similarly, Demchenko *et al.* synthesized PLA–Ag–PEI nanocomposites with significant antiviral and antimicrobial activity using a thermochemical reduction of Ag^+^ ions within polylactide films.^84^ Despite potential, AgNP–PNCs face challenges: toxicity risks vary by AgNP size, shape, and concentration; agglomeration or degradation can affect stability; production costs are high; regulatory approval can be lengthy; efficacy varies by virus type; and proper storage is crucial to maintain antiviral effectiveness. Addressing these issues is key for clinical application.

#### 8.1.3. Antifungal medical material

Antifungal materials are vital in biomedical applications, preventing fungal infections that threaten immunocompromised patients. These materials protect biomedical devices by inhibiting fungal growth, enhancing durability and reducing dependency on antifungal medications. Their role is essential in maintaining patient safety and improving the efficacy of biomedical applications, making ongoing research crucial for advancing patient care. Ferreira *et al.* synthesized non-cytotoxic AgNPs–DDT/PBAT nanocomposites with antifungal properties through a reduction/precipitation method, incorporating AgNPs at 0.25, 0.5, and 2 wt%.^85^ These nanocomposites demonstrated improved mechanical performance and antifungal activity, suggesting potential in biomedical applications. Further research, expert oversight, and regulatory compliance are recommended to ensure safe, effective use of these nanocomposites in biomedical materials. Table 4 summarizes the recent applications of AgNP–PNCs for antimicrobial purposes.

**Table 4:** Summary of the recent applications of AgNP–PNCs for antimicrobial purposes

Metal particles	Polymer matrix	Supporting materials	Purpose	Ref.
AgNP	Poly(hydroxymethyl 3,4-ethylenedioxythiophene)	Conjugated PNCs	Medical implant antimicrobial coating	86
	Polystyrene			
	(3-Aminopropyl)triethoxysilane			
AgNP	Chitosan		Antifungal	87
AgNP	Chitosan		Antibacterial, antibiofilm, antifungal, antioxidant and wound-healing activities	88
AgNP	Polysulfone (PSF), polyimide (PI) or polyetherimide (PEI)	Zeolite crystals	Antifungal	89
AgNP	Montmorillonite		Antifungal	90
AgNP	Acrylic latexes		Antifungal	91
AgNP	PDMS	Lecithin and montmorillonite	Antibacterial	92
AgNP	PLA	Polyethyleneimine (PEI)	Antimicrobial and antiviral	93
AgNP		GO	Antimicrobial	94

### 8.2. Tissue engineering

AgNP–PNCs show strong potential in tissue engineering, offering antimicrobial properties that prevent implant infections while enhancing mechanical strength and electrical conductivity. Their polymer matrices add biocompatibility and controlled degradation, useful for applications like wound healing, bone regeneration, and implants.^95^ Hosseini *et al.* synthesized polyaniline (PANI) in bacterial cellulose/AgNP hydrogels with rose-like PANI morphologies, achieving high porosity (80%) and enhanced modulus (*G*′) under specific synthesis conditions.^96^ Zulkifli's team developed hydroxyethyl cellulose/AgNP scaffolds for skin tissue, showing high porosity, moderate degradation, and low cytotoxicity with hFB cells, supporting their potential as skin substitutes.^97^ Marsich engineered antibacterial alginate/hydroxyapatite scaffolds with AgNPs for bone grafts, achieving a 341.5 μm pore size and effective osteoblast proliferation with antimicrobial activity, highlighting their promise for tissue engineering.^98^Table 5 summarizes the recent applications of AgNP–PNCs for tissue engineering purposes.

**Table 5:** Summary of the recent applications of AgNP–PNCs for tissue engineering purposes

Metal particles	Polymer matrix	Supporting materials	Purpose	Ref.
Ag	Hydroxyethyl cellulose (HEC)		Implant and skin tissue engineering	99
Ag	Chitosan, carboxymethyl cellulose	Cellulose nanowhiskers	Bone tissue engineering	95
Ag	Starch and gelatin	Bioactive glass particles (BG)	Bone tissue engineering and antibacterial	100
Ag	Bacterial cellulose, polyaniline, PEG	Nanocomposite aerogel	Soft tissue engineering and antibacterial	96
Ag	Agarose, chitosan		Soft tissue engineering and antibacterial	101

### 8.3. Wound dressings and tissue scaffolds

AgNPs are highly valued in wound dressings and tissue scaffolds for their antibacterial, biocompatible, and tissue-regenerative properties. Integrating AgNPs into PNCs has shown significant efficacy in reducing bacterial growth and accelerating wound healing, with promising applications in tissue regeneration.^51^ However, further research is needed to confirm long-term biocompatibility and safety. Mehrabani *et al.* created silk fibroin/chitin nanocomposite scaffolds with AgNPs (0.001–0.1%), achieving strong antimicrobial activity, porosity, and biocompatibility, suitable for wound dressings.^102^ Additionally, cellulose nanofiber (CNF) films with AgNPs derived from *Cassia alata* demonstrated mechanical strength, water absorption, and potential anti-diabetic effects, suggesting a cost-effective wound-healing material.^103^ Ediyilyam *et al.* fabricated biogenic AgNP–chitosan scaffolds *via* lyophilization, using *Mussaenda frondosa* for synthesis. These biocompatible scaffolds exhibited enhanced biological and physical properties, showing potential for broader biomedical applications.^104^Table 6 summarizes the recent applications of AgNP–PNCs for wound dressing purposes.

**Table 6:** Summary of the recent applications of AgNP–PNCs for wound dressing purposes

Metal particles	Polymer matrix	Supporting materials	Purpose	Ref.
Ag	Collagen	Catechin	Second degree infected burns	105
			Angiogenic	
			Antibacterial	
AgNP	Chitosan	Pectin	Wound healing	106
Ag	Starch	Nanocomposites hydrogel membranes	Wound dressing	107
	PVA		Antimicrobial	
Ag	Chitosan		Antibacterial antibiofilm and wound healing	88
Ag	Sodium alginate	Nanocomposites hydrogel		108
	Ethylene glycol			
	Acrylic acid			
Ag	Cellulose acetate	GO modified nanofibrous mats	Antimicrobial performance and cutaneous wound healing	109
	PU			
AgNP	PVA	Xanthan gum	Dermal dressings antibacterial wound healing	110
	Sodium carboxymethyl cellulose	Hypromellose		
AgO	Carboxymethyl chitosan	Tragacanthin gum	Wound dressing	111
TiO_2_				
Ag_2_O	Cellulose acetate	GO	Antibacterial wound healing	112
ZnS				

### 8.4. Drug delivery systems

**Fig. 6:**
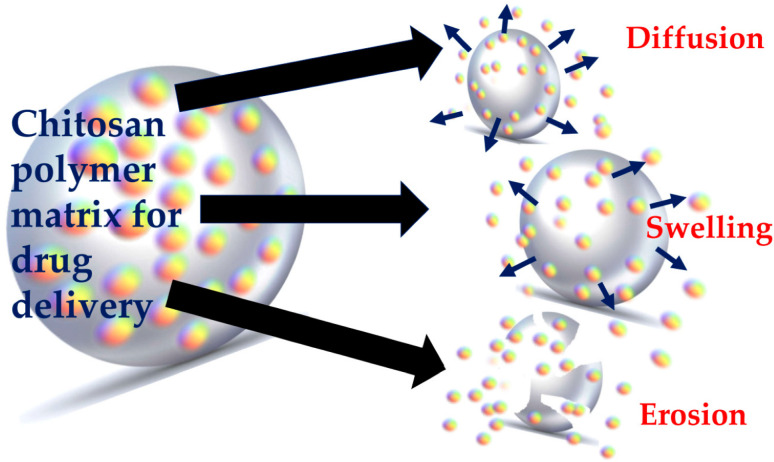
The proposed mechanism of predominant three processes involved in polymer nanocomposite-based drug delivery system. Reproduced with permission.^114^ Copyright 2021 by the authors. Licensee MDPI Basel Switzerland.

AgNP–PNCs show strong potential in drug delivery systems, combining a polymer matrix with AgNPs. The high surface area and antimicrobial properties of AgNPs enhance drug solubility, controlled release, and targeted delivery to specific cells or tissues. The polymer matrix provides stability, while AgNPs improve drug loading and release efficiency.^113^ These nanocomposites promise advancements in drug delivery by offering enhanced solubility, controlled release, and precise targeting, though further research is needed to confirm their clinical safety. The proposed mechanism, including three main processes, is illustrated in Fig. 6.

**Fig. 7:**
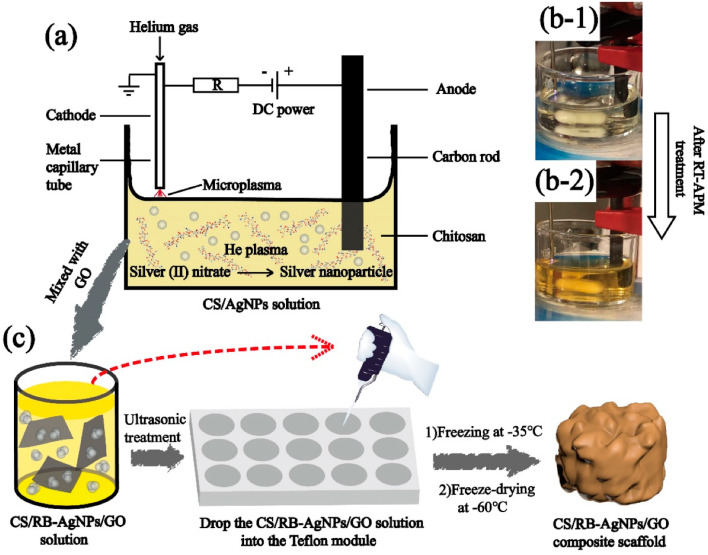
(a) The schematic diagram of the atmospheric pressure microplasma setup for the preparation of CS/AgNPs (b) CS + AgNO_3_ solution before and after RT-APM treatment and (c) the preparation of CS/RB-AgNPs/GO nanocomposite using a freeze-drying process. Reproduced with permission.^115^ Copyright 2021 by the authors. Licensee MDPI Basel Switzerland.

Su *et al.* developed a chitosan–AgNP nanocomposite with a raspberry-like morphology combined with GO, using a microplasma process at room temperature and atmospheric pressure for efficient AgNP incorporation (Fig. 7).^115^ This pH-sensitive composite enables single or dual drug release: at pH 7.4, methyl blue released quickly in the first 10 hours, reaching 70–80% after 40–50 hours, while fluorescein sodium achieved a 45% release over 240 hours. The dual system slowed methyl blue release, with both rates accelerating at pH 4. The composite also showed strong antibacterial effects against *E. coli* and *S. aureus*, along with efficient photothermal conversion under near-infrared lasers, making it suitable for applications in healthcare, water treatment, and antimicrobial use.

Yadollahi *et al.* explored chitosan hydrogel beads crosslinked with sodium tripolyphosphate to encapsulate AgNPs for drug delivery.^116^ X-ray diffraction and SEM confirmed AgNP integration, with the beads exhibiting strong antibacterial effects against *E. coli* and *S. aureus* and enhanced swelling capacity. *In vitro* studies showed that AgNPs facilitated sustained drug release, increasing with higher AgNP concentrations, positioning these PNC beads as a promising drug delivery system. Neri *et al.* examined the release of silibinin, a liver-protective drug, from a PEG–PLA@Ag composite, where AgNPs responded to specific laser wavelengths *via* Surface Plasmon Resonance (SPR), generating localized heat for controlled drug release. This system's photothermal properties show potential for targeted drug delivery and photothermal therapies.^117^ Yang *et al.* developed C-β-CD-modified AgNPs with quaternary ammonium and amino groups for drug delivery.^118^ Thymol was loaded into this cyclodextrin–AgNP composite, with C-β-CD acting as both a reducing agent and stabilizer. The successful synthesis, confirmed by XRD, UV-vis, FT-IR, and ^1^H-NMR, showed effective dispersion and aggregation resistance. HS-SPME-GC-MS and UV-vis spectroscopy verified drug-loading efficiency, with a noted increase in particle size after drug loading. Table 7 summarizes the recent applications of AgNP–PNCs for drug delivery system.

**Table 7:** Summary of the recent applications of AgNP–PNCs for drug delivery systems

Metal particles	Polymer matrix	Supporting materials	Purpose	Ref.
Ag	Chitosan	Nanochitosan polymer composite	Drug delivery	114
Ag	Carboxymethylcellulose, poly(acrylamide)	Nanocomposite hydrogel	pH sensitive drug delivery	119
AgNP	Tragacanth gum	Nanocomposite hydrogel	Drug delivery and inactivation of multidrug-resistant bacteria	120

### 8.5. Biosensors for disease diagnosis

**Fig. 8:**
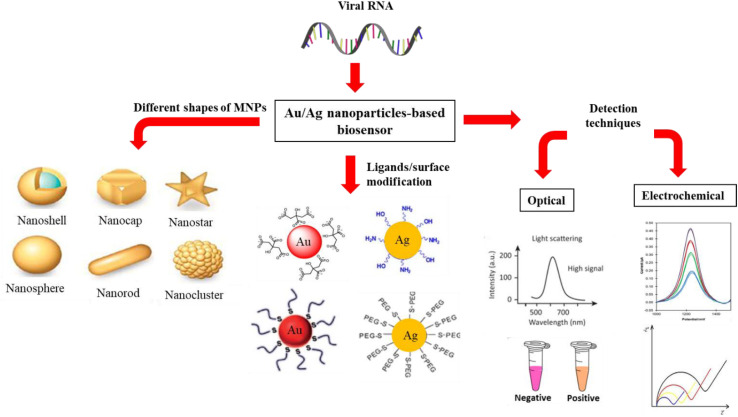
A graphical representation to depict the various forms of MNPs (such as AuNPs or AgNPs) and the use of ligands to stabilize them. The optical and electrochemical characteristics of the MNPs were examined using techniques for optical and electrochemical sensing confirming their properties. Reproduced with permission.^122^ Copyright 2021 by the authors. Licensee MDPI Basel Switzerland.

AgNP–PNCs hold great potential for disease diagnosis biosensors due to AgNPs' high surface area and signal amplification, making them ideal for biosensing.^121,122^ As shown in Fig. 8, PNCs with metallic nanoparticles like Ag and Au are well-suited for these applications. The polymer matrix in these composites provides stability, while AgNPs enhance sensitivity and specificity, which are useful for detecting cancer biomarkers, infectious diseases, and cardiovascular markers, as well as small molecules like drugs and toxins. AgNP–PNCs can be tailored to specific biosensing needs, with applications for electrochemical and optical sensors. Ananth *et al.* developed AgNP biosensors coated with BSA and PVA, showing wavelength shifts and no behavioral toxicity in a mouse model, indicating potential in examining cellular processes.^121^ Yazdanparast *et al.* introduced a biosensor for detecting MCF-7 breast cancer cells using an aptamer-based AgNP assay, achieving high selectivity and a detection limit of 25 cells, suitable for early breast cancer detection.^123^

### 8.6. Anticancer agents and cancer therapy

AgNP–PNCs show promise as anticancer agents, leveraging AgNPs' cell-killing and antimicrobial properties, with the polymer matrix providing stability and biocompatibility. These composites enable targeted drug delivery and can act as photothermal agents, where AgNPs generate heat upon near-infrared activation to destroy cancer cells. AgNP–PNCs also enhance chemotherapy by boosting drug uptake and reducing side effects, potentially preventing drug resistance.^124^ Senthilkumar *et al.* developed a PDMA/Ag nanocomposite using a chemical oxidative method, achieving a high AgNPs content (42.1%) with spherical particles around 25 nm.^125^ The PDMA/Ag composite showed strong antibacterial properties and significantly reduced cell viability in HeLa cancer cells compared to PDMA alone. Haghparasti *et al.* synthesized AgNPs with white tea extract, forming a Wt/Ag@Mt nanocomposite with antioxidant properties. The Wt/Ag@Mt-NCs displayed a crystalline structure and effective cytotoxicity against MOLT-4 cells, with an IC_50_ of 0.0039 μM, outperforming drugs like doxorubicin and cisplatin. These bio-synthesized nanoparticles hold promise for pharmaceutical applications, offering an environmentally sustainable approach.^126^

### 8.7. Dental materials for caries and periodontal disease prevention

AgNPs have been widely explored for their antimicrobial potential and applications in dentistry. Integrating AgNPs into PNCs enhances the mechanical, physical, and antibacterial properties of dental materials, aiding in dental imaging, where their optical properties improve contrast in X-rays, aiding cavity and periodontal disease detection.^127–129^ AgNPs are particularly effective against bacteria causing dental caries and periodontal disease; when embedded in dental composites and coatings, they inhibit bacterial growth more effectively than traditional materials. This efficacy is due to AgNPs' small size, allowing them to penetrate bacterial cells and disrupt functions, while the polymer matrix ensures even distribution throughout the material. To address the need for low-cost, user-friendly detection of dental caries and periodontal diseases, Ma *et al.* developed a wearable mouthguard with Au@Ag nanorods and PDMS, which detects lesion sites by a color change due to H_2_S gas from bacterial decay.^127^ The mouthguard is mechanically robust, chemically stable, biocompatible, and accurately locates lesion sites, showing promise for self-monitoring oral health and targeted treatment. Table 8 summarizes the recent applications of AgNP–PNCs for dental materials and treatment.

**Table 8:** Summary of the recent applications of AgNP–PNCs for dental materials and treatment

Metal particles	Polymer matrix	Supporting materials	Purpose	Ref.
AgNPs	Polydopamine (PDA)	Hydroxyapatite nanowires	Long-term clinical restorations in dental treatment	128
AgNPs	Resin	Halloysite nanotubes (HNT)	Restorative dental material for patients with high risk of dental caries	129
AgNP	Acrylic resin	Sodium citrate	Antimicrobial effects against *C. albicans* biofilm	130
			Dental treatment	
AgNP	Gelatin	Halloysite nanotubes (HNT)	Dental prostheses	131
	PMMA			
AgNP	PMMA	Boron nitride nanosheets (h-BNNs)	Oral denture bases	132

### 8.8. Other biomedical applications

AgNP–PNCs have emerged as promising candidates for various biomedical applications, including optical materials such as contact lenses, orthopedic implants, neural interfaces, medical textile coatings, skin protection, cosmetics, and anti-inflammatory products. The diverse biomedical uses of AgNP–PNCs are comprehensively summarized in Table 9.

**Table 9:** Summary of the recent multipurpose biomedical applications of AgNP–PNCs

Metal particles	Polymer matrix	Supporting materials	Purpose	Reference
AgNPs	PVA	PNC hydrogel	Sensing devices cosmetic	133
	Poly(vinyl pyrrolidone)		Medical contact lenses	
AgNPs	Polyvinyl-pyrrolidone (PVP)	Poly(2-hydroxyethyl methacrylate) (pHEMA)	Contact lens	134
AuNPs				
Spherical AgNPs	pHEMA		Contact lens blue-yellow color vision deficiency	135
AgNP	Arabinoxylan-*co*-acrylic acid	GO	Orthopedic implant	136
Al_2_O_3_NP	Nano-hydroxyapatite (nHAp)			
AgNP Ti	Amorphous hydrocarbon	Nanocomposite coating	Orthopedic implant	137
AgNP Ti		Nanocomposite Ag layer	Orthopedic implant	138
Ag nanowires	Poly(ε-decalactone) (EDL)		Electrically conducting neural interface biomaterial	139
Ag/Au nanowires	Poly(3-methoxypropyl acrylate) (PMC3A)		Long-term implantable flexible and transparent neural interface	140
Au nanoshell	PDMS	Tough stretchable and self-healing polymer	Neuroprosthetics for *in vivo* bidirectional signaling	141
AgNP	Methylenebis(phenyl urea) (MPU)			
	Isophorone bisurea units (IU)			
Silver nanowires (AgNW)	Poly(ε-decalactone)	CNT	Neural interface materials	142
	Poly(hydroxymethyl 3,4-ethylenedioxythiophene) microspheres (MSP)			
AgNP	Carboxymethyl chitosan	Cotton fabric	Antibacterial and antifungal textile coating	143
AgNP	Chitosan	One-pot microwave synthesized PNC	Antibacterial antioxidant wound healing	144
	Polyethylene oxide			
Photothermal AgNPs	Hyaluronic acid	Tannic acids (TA)	Antibacterial and antioxidant	145
Au, Ag and ZnO NPs	PVA	MMT clay	Skin protection cosmetics UV protection coating	146
	Starch			
AgNP	Poly(amino acid)	Nanocomposite fibrous mesh	Anti-inflammatory drug	147

## 9. Challenges and solutions for successful biomedical application of AgNP–PNCs

The successful translation of AgNP–PNCs into biomedical applications requires addressing key biocompatibility, stability, cost, standardization, and regulatory hurdles. While AgNP–PNCs hold great promise in infection control, drug delivery, and implantable medical devices, challenges related to toxicity, stability, large-scale production, and regulatory compliance must be resolved to facilitate clinical adoption. This section presents current challenges along with strategic solutions to enhance the real-world application of AgNP–PNCs in biomedical fields.

### 9.1. Biocompatibility and cytotoxicity concerns

AgNPs exhibit size- and dose-dependent toxicity, which can induce oxidative stress, DNA damage, mitochondrial dysfunction, and inflammatory responses in human cells, limiting their safe use in biomedical applications.^148^ The mechanism of toxicity primarily involves the generation of ROS, disruption of cellular membranes, and interference with protein function. The immune response triggered by AgNP exposure remains a critical issue, requiring further investigation into inflammatory pathways and potential long-term effects. To enhance biocompatibility, AgNPs can be functionalized with biocompatible coatings, such as PEG, chitosan, or albumin, which minimize direct AgNP–cell interactions and reduce inflammatory responses.^149^ Hybrid nanostructures, such as AgNPs embedded in lipid or polymeric nanocarriers, provide a controlled release mechanism that mitigates acute toxicity while preserving therapeutic effects. Surface engineering techniques, including biomimetic coatings, can improve AgNP compatibility with biological environments. Preclinical evaluation through advanced organ-on-a-chip models and 3D cell cultures will provide more accurate predictions of AgNP–PNC biocompatibility before *in vivo* testing.

### 9.2. Stability challenges in biological environments

AgNP–PNC stability is a critical factor influencing long-term efficacy and safety in biomedical applications. The tendency of AgNPs to aggregate, oxidize, and release excess silver ions in physiological conditions can significantly impact their effectiveness.^150^ Key factors affecting stability include pH variations, ionic strength, temperature fluctuations, and interactions with biological macromolecules. To counteract these challenges, surface modifications using stabilizing agents such as PEG, surfactants (SDS, CTAB, PVP), and natural polymers (chitosan, alginate, or dextran) have been shown to prevent aggregation and enhance dispersion in biological media. Incorporating AgNPs into a crosslinked polymeric network improves nanoparticle stability by restricting their movement and controlling silver ion release. Hybrid AgNP-based systems, such as AgNP-loaded mesoporous silica nanoparticles or graphene-based nanocomposites, offer improved chemical stability and prolonged antimicrobial activity, making them promising candidates for long-term medical implants and wound dressings.

### 9.3. Cost constraints and scalable production

The large-scale production of AgNP–PNCs remains expensive due to high silver consumption, energy-intensive synthesis processes, and the need for advanced functionalization techniques. Traditional chemical synthesis methods require toxic reducing agents, which complicate purification steps and increase production costs. To make AgNP–PNCs more commercially viable, cost-effective and sustainable synthesis approaches have been explored. Green synthesis techniques, utilizing plant extracts, microbial cultures, or biomolecules as reducing and stabilizing agents, have demonstrated promise in producing AgNPs with minimal environmental impact and reduced production costs.^151^ Microwave-assisted synthesis has emerged as a rapid and energy-efficient method that ensures controlled AgNP growth with minimal precursor wastage. Adopting scalable microfluidic reactors allows for continuous synthesis, ensuring batch-to-batch consistency and optimized nanoparticle characteristics. Advances in automated in-line characterization will enhance production efficiency, enabling real-time monitoring of AgNP properties during synthesis.

### 9.4. Standardization issues in AgNP–PNC fabrication

The absence of standardized protocols in AgNP–PNC synthesis leads to inconsistencies in particle size, shape, surface charge, and stability, affecting reproducibility and clinical reliability. Variability in nanoparticle formulations presents significant challenges for regulatory approval and large-scale manufacturing. To address these challenges, it is essential to establish standardized synthesis and characterization protocols that define optimal nanoparticle physicochemical properties for biomedical applications. Characterization techniques such as dynamic light scattering (DLS), transmission electron microscopy (TEM), zeta potential analysis, and X-ray photoelectron spectroscopy (XPS) should be employed systematically to ensure batch consistency. Regulatory bodies such as the International Organization for Standardization (ISO) and the National Institute of Standards and Technology (NIST) are working toward defining standard measurement frameworks for nanomaterials. Establishing reference AgNP materials for biomedical applications will facilitate cross-laboratory comparisons and regulatory compliance.

### 9.5. Regulatory approval and clinical translation

AgNP-based materials face regulatory barriers due to concerns over nanoparticle toxicity, environmental persistence, and potential long-term effects on human health. Regulatory agencies such as the U.S. Food and Drug Administration (FDA), European Medicines Agency (EMA), and World Health Organization (WHO) require comprehensive preclinical and clinical evaluations before approving AgNP–PNCs for medical applications. Currently, FDA-approved AgNP-based products are limited, with most applications restricted to topical antimicrobials, wound dressings, and medical coatings. For systemic applications, including drug delivery and injectable nanomedicines, extensive pharmacokinetic and toxicological assessments are required to evaluate silver biodistribution, clearance rates, and immunogenicity. Compliance with Good Manufacturing Practices (GMP) and ISO nanotechnology standards will be essential for commercializing AgNP–PNCs in clinical settings. To facilitate regulatory approval, collaborative efforts between academia, industry, and regulatory agencies are needed to develop standardized safety assessment frameworks. Risk-benefit analyses, focusing on the therapeutic advantages of AgNPs *versus* potential long-term risks, will be vital in shaping future regulatory guidelines.

### 9.6. Comparison with alternative antimicrobial nanomaterials

While AgNPs are among the most effective antimicrobial agents, concerns about toxicity, environmental impact, and potential bacterial resistance have led researchers to explore alternative nanomaterials with comparable efficacy and lower cytotoxicity. ZnO, copper nanoparticles (CuNPs), TiO_2_, and carbon-based nanomaterials such as GO have demonstrated broad-spectrum antimicrobial properties with minimal adverse effects on mammalian cells. ZnO nanoparticles exhibit photoactivated antimicrobial properties, generating ROS under UV exposure, making them ideal for antimicrobial coatings and wound dressings. CuNPs provide effective bacterial inhibition, though their higher cytotoxicity and oxidative stress potential require careful dose optimization. TiO_2_, often combined with AgNPs, enhances photodynamic antibacterial therapy, offering a synergistic approach to infection control. Carbon-based materials, including GO and CNTs, exhibit membrane-disrupting antibacterial mechanisms, reducing the risk of resistance development. A comparative evaluation of AgNP–PNCs with alternative antimicrobial nanomaterials will provide deeper insights into material selection for specific biomedical applications, ensuring optimal efficacy with minimal side effects. Future research should explore hybrid nanostructures, integrating AgNPs with other nanomaterials to create multifunctional antimicrobial coatings with controlled release capabilities.

## 10. Future perspective of AgNP–PNCs for biomedical applications

The potential of AgNP–PNCs in biomedical applications continues to be a promising field of study. The unique properties of AgNPs, including antimicrobial activity, high thermal and electrical conductivity, and enhanced mechanical properties, when integrated with polymer matrices, create innovative materials suited for various medical applications. However, the full realization of these materials in clinical settings will depend on advancements across several fronts. As research progresses, the biocompatibility of AgNP–PNCs remains a primary concern. Innovations in surface modification and functionalization of nanoparticles to reduce cytotoxicity while maintaining antimicrobial efficacy will be crucial. Future studies must establish robust biocompatibility profiles through comprehensive *in vitro* and *in vivo* assessments.^152^

Developing cost-effective, scalable manufacturing processes that can produce high-quality AgNP–PNCs consistently is essential. Techniques like 3D printing and automated synthesis could revolutionize the production of these nanocomposites, making them more accessible for widespread medical use.^153,154^ Darbandi *et al.* reported significant advancements in the fabrication of 3D-printed dental restorations using resins modified with zirconium dioxide (ZrO_2_) nanoparticles and AgNP-immobilized halloysite nanotubes (HNC/Ag).^154^ Their study highlights the potential of these nanoparticles to enhance the mechanical properties of 3D-printed resins, which is crucial for biomedical applications. They evaluated the impact of different mass fractions of ZrO_2_ and HNC/Ag on the flexural strength, flexural modulus, fracture toughness, and microhardness of the resins. The optimal ratios identified were ZrO_2_ at 4% and HNC/Ag at 5%, which significantly increased the flexural strength, flexural modulus, and fracture toughness of the resin. Moreover, higher concentrations of ZrO_2_ (up to 16%) and HNC/Ag (up to 7.5%) were found to improve the hardness of the resin. The study underscores the potential of advanced manufacturing techniques in developing high-performance, biocompatible materials for biomedical applications. By leveraging the properties of ZrO_2_ and HNC/Ag nanoparticles, it is possible to create dental restorations with superior mechanical and physical characteristics, paving the way for more durable and practical biomedical devices. Future research could explore the integration of these nanoparticles into other types of resins and their applications in various biomedical fields beyond dentistry. The use of 3D printing technology, combined with advanced nanoparticle modifications, holds promise for developing innovative and highly functional biomedical materials.

AgNP–PNCs show promise in targeted drug delivery applications. The ability to engineer nanoparticles that can navigate to specific tissue sites and release therapeutic agents in a controlled manner is an area ripe for development. Future research could focus on enhancing the targeting capabilities of these systems, possibly using ligand-directed strategies.^155^ The optical properties of AgNPs can be exploited to improve diagnostic imaging technologies. AgNP–PNCs could be designed to enhance contrast in imaging modalities such as MRI or ultrasound, providing clearer, more precise images that could aid in the early detection of diseases. As with any emerging medical technology, regulatory challenges must be navigated to ensure safety and efficacy before clinical adoption. Establishing standardized protocols for the testing, use, and disposal of AgNP–PNCs will be necessary. Additionally, ethical considerations, particularly concerning long-term effects and environmental impact, will require ongoing dialogue among scientists, regulators, and the public.

While current applications are promising, exploring AgNP–PNCs in regenerative medicine, neural interfaces, and wearable health monitors could provide new avenues for treatment and patient care. Research into these applications could open up novel therapeutic avenues and potentially revolutionize aspects of healthcare. In the face of rising antibiotic resistance, AgNP–PNCs could offer a potent alternative due to their broad-spectrum antimicrobial properties. Future research might optimize these properties to create surfaces and devices resistant to colonization by pathogenic microbes, thus reducing the incidence of hospital-acquired infections. The future of AgNP–PNCs in biomedical applications looks promising, with potential breakthroughs spanning various medical fields. The interdisciplinary nature of this research, involving materials science, nanotechnology, medicine, and engineering, suggests a collaborative approach will be pivotal. Continuous evaluation and adaptation to emerging challenges and innovations will be key to its success as technology matures.

## 11. Conclusion

AgNP–PNCs represent a promising frontier in biomedical materials, combining the antimicrobial, optical, and conductive properties of AgNPs with the versatility, stability, and biocompatibility of polymers. These nanocomposites have demonstrated exceptional potential across various medical applications, from antimicrobial coatings for medical devices to advanced wound dressings, tissue engineering scaffolds, and drug delivery systems. The development and optimization of AgNP–PNCs have significantly enhanced the functionality of these materials, making them highly suitable for addressing critical health challenges and improving patient outcomes. Despite these advances, challenges remain. The biocompatibility and cytotoxicity of AgNPs, dependent on particle size, shape, and concentration, are essential to ensure safe clinical applications. Moreover, standardizing synthesis methods and improving scalability are vital for adopting AgNP–PNCs widely in commercial and clinical settings. Addressing these factors will facilitate regulatory approvals and streamline the integration of AgNP–PNCs into various biomedical fields.

This review has comprehensively analyzed the current landscape, covering recent advancements in synthesis and functionalization, innovative applications, and future research directions for AgNP–PNCs. By fostering interdisciplinary research and collaboration, the field is poised to overcome present limitations and unlock the full potential of AgNP–PNCs. As research progresses, these nanocomposites could revolutionize medical materials, offering safer, more effective solutions for various biomedical applications.

## Data availability

No primary research results, software or code have been included and no new data were generated or analyzed as part of this review.

## Author contributions

Mohammad Harun-Ur-Rashid: conceptualization, data curation, formal analysis, writing original draft, and review. Tahmina Foyez: data curation, formal analysis, writing original draft, and review. Suresh Babu Naidu Krishna: formal analysis, data curation, resources, visualization, writing-review, and editing. Sudhakar Poda: data curation, formal analysis, validation. Abu Bin Imran: conceptualization, supervision, data curation, visualization, writing original draft, writing review and editing.

## Conflicts of interest

All authors certify that they have no affiliations with or involvement in any organization or entity with any financial interest or non-financial interest in the subject matter or materials discussed in this manuscript.
